# NLRC3 High Expression Represents a Novel Predictor for Positive Overall Survival Correlated With CCL5 and CXCL9 in HCC Patients

**DOI:** 10.3389/fonc.2022.815326

**Published:** 2022-01-25

**Authors:** Chengpan Wang, Jieyi Shi, Jietian Xu, Qiaoyu Fu, Youpeng Ding, Jessie Yang, Binbin Liu, Qiang Gao, Jie Qin, Chunmin Liang

**Affiliations:** ^1^ Lab of Tumor Immunology, Department of Human Anatomy, Histology and Embryology, Basic Medical School of Fudan University, Shanghai, China; ^2^ Department of Liver Surgery and Transplantation, Liver Cancer Institute, Zhongshan Hospital, Fudan University, and Key Laboratory of Carcinogenesis and Cancer Invasion of Ministry of Education, Shanghai, China

**Keywords:** NLRC3, CCL5, CXCL9, overal survival, hepatocellular carcinoma, CD8+ T cell

## Abstract

NLRC3 (NLR family caspase recruitment domain containing 3) has been reported as a factor of inhibiting inflammatory responses. It’s role in HCC (hepatocellular carcinoma) is still unknown. In this study we firstly used the GEO (Gene Expression Omnibus) database and mIHC (multiple immunohistochemical analysis) with TMAs (tumor tissue microarrays) of HCC patients to evaluate NLRC3 levels. The tumor-bearing mouse models were also established with NLRC3 over-expressing and knock-down Hepal-6 cells to assess its effect. The data showed high NLRC3 expression was related with favorable overall survival (*P*=0.0386) and disease-free survival (*P*=0.0458). In addition, NLRC3 expression showed a positive correlation between CD8^+^ T cells infiltration. *In vivo*, NLRC3-overexpressing Hepal-6 tumors showed increased CD8^+^ T cell infiltration. NLRC3-knockdown Hepa1-6 tumors displayed decreased CD8^+^ T cell infiltration. At the same time, we also found the positive correlations between NLRC3 and CCL5 (C-C motif chemokine ligand 5, *P*<0.0001, R^2^ = 0.2372) as well as CXCL9 (C-X-C motif chemokine ligand 9, *P*<0.0001, R^2^ = 0.2338) expressions. So NLRC3 high expression represents a novel predictor for positive survival outcomes in HCC patients, and NLRC3 is involved in CD8^+^ T cell infiltration, which is correlated with increased CCL5 and CXCL9 in TME (tumor microenvironment). This study implies that boosting NLRC3 is a promising treatment to enhance survival in HCC patients.

## Introduction

According to the latest data survey, HCC (hepatocellular carcinoma) ranks the second-highest mortality of all tumors in the world (1). Although new diagnostic and therapeutic techniques have been developed (2, 3), HCC outcomes still tend to be poor because of significant recurrence and metastasis (4, 5), as well as absence of new promising biomarkers (6). In addition, up to 80% of HCCs occur after chronic liver diseases such as hepatitis and over 90% of cases are associated with chronic liver disease, suggesting that HCC occur as a result of inflammation (7). Inflammation is associated with cancer and appears to responsible for various aspects of tumor development (8). An inflammatory TME (tumor microenvironment) is formed by the interactions of tumor cells, stromal cells, and inflammatory cells (9), so the identification of potential biomarkers related with inflammation is assumed its priority especially in HCC study.

NLRC3 (NLR family caspase recruitment domain containing 3) has been reported as a factor of inhibiting inflammatory responses. It was previously reported that NLRC3 prevents the growth of colorectal cancer by inhibiting PI3K-mTOR (mammalian target of rapamycin) pathway (10). CD8^+^ T cell-targeting therapy has also been proved highly effective (11). So, we suppose NLRC3 maybe links with CD8^+^ T cell infiltration in HCC.

In this study, we firstly analyzed the relationship between the expression of NLRC3 mRNA and outcomes of HCC patients using publicly available databases. Then we verified this relationship in clinical 211 HCC patients by IHC analysis of tumor tissue microarrays (TMAs). Followed, we explored the association between the expression of NLRC3 levels and CD8^+^ T cell infiltration with mIHC. At last, we established tumor-bearing mouse models *in vivo* to determine the effect of NLRC3 on CD8^+^ T cell infiltration by over-expressing or knocking-down the NLRC3 gene. Furthermore, we investigated the chemokines that could recruit T cells to tumor, and their relationships with NLRC3 in HCC.

## Materials and Methods

### Clinical Samples

Tissue microarray (TMA), including 211 paired tumor and peritumor tissues, was obtained from Zhongshan Hospital Affiliated to Fudan University (Shanghai, China). All patients were confirmed as HCC by two pathologists independently and underwent complete surgical excision between January 2006 and August 2006 as described in our previous study (12). The study was approved by the Clinical Research Ethics Committee of Zhongshan Hospital and informed consent was obtained from each patient.

### Cell Line

A murine hepatoma cell line (Hepa1-6) was cultured in DMEM (Dulbecco’s modified eagle medium) containing 10% fetal bovine serum (FBS, Sigma-Aldrich, St Louis, MO, USA). Cells were transfected with the pLVX-IRES-Puro plasmid encoding full-length mouse NLRC3 for overexpression (Hepa1-6-NLRC3). Blank pLVX-IRES-Puro plasmid was used as vector control. The NLRC3 knockdown in Hepa1-6 (Hepa1-6-shRNA1) was produced by transfection with pLVX-shRNA1-Puro. The siRNA target sequences were as follows: siRNA-1: GAGATCCAGGTAGACCACCTGAT-CA and siRNA-2: CCAGAAATTTCTGTCTGGATCACCT.

### Animals

Adult male C57BL/6N mice were used for experiments at 4-6 week of age and purchased from Vital River Laboratory (Beijing). All mice housed in environmentally controlled conditions with a 12-hr light/dark cycle (lights on at 7:00 am) with ad libitum food and water. Animals were allocated randomly into different groups where appropriate. All procedures were conducted in accordance with the Animal Care and Use Committee of School of Basic Medical Sciences of Fudan University.

C57BL/6N mice were divided into four groups: (1) Hepa1-6-shRNA1 group (n=5), each mouse injected into the lower back with 5 × 10^6^ Hepa1-6 cells threated with NLRC3 siRNA; (2) Hepa1-6-sh-NC group (n=5), each mouse injected into the lower back with 5 × 10^6^ Hepa1-6 cells threated with siRNA control; (3) Hepa1-6-vector group (n=5), each mouse injected with 5 × 10^6^ blank vector infected Hepa1-6 cells; (4) Hepa1-6-NLRC3 group (n=5), each mouse injected into the lower back with 5 × 10^6^ NLRC3 overexpressed Hepa1-6 cells. After 16 days, the mice were sacrificed and the tumor tissues were extracted.

### Quantitative Real-Time Polymerase Chain Reaction

The real-time quantitative polymerase chain reaction (RT-PCR) was conducted according to previously described methods (13). GAPDH was used as an internal control. The NLRC3 primers were 5’-GTC AGC TGC TAC AAG TCC GGG AC-3’(forward) and 5’-GAG CCT CAG AGT GCT TCG GTA TCC-3’(reverse). GAPDH primers were 5’-AGG TTG TCT CCT GCG ACT TCA -3’(forward) and 5’-TGG TCC AGG GTT TCT TAC TCC-3’(reverse). Data semiquantitative analysis used the 2^−ΔΔCt^ method. Glyceraldehyde‐3‐phosphatede hydrogenase (GAPDH) served as an internal standard. Hepa1-6-sh-NC and Hepa1-6-Vector as the negative control groups for Hepa1-6-shRNA1 and Hepa1-6-NLRC3 respectively.

### Immunohistochemistry

Immunohistochemistry (IHC) was applied to tissue microarrays (TMAs) as described in our previous study (12). The semi-quantitative scoring categories were negative (0), weak (1), moderate (2), and strong (3) while the percentage of staining was classified as 1 (0-25%), 2 (>25-50%), 3 (>50-75%) or 4 (>75-100%) by two observers independently. The sum of these values was used to categorize NLRC3 levels as either low (0-3) or high (4-7).

### Multiple Immunohistochemistry

Multiple immunohistochemistry (mIHC) was conducted by the Opal method on TMAs. Tissues were incubated with antibodies: rabbit anti-NLRC3 antibody (Origene, USA), rabbit anti-CD8 antibody (Abcam, UK), rabbit anti-CD11c antibody (Abcam, UK), et al. After a subsequent antigen retrieval, the nuclei were counterstained with DAPI at room temperature in the dark for 30 minutes, and then scanned using a multispectral Vectra scanner and quantitative imaging system (Olympus VS120, Japan).

### Flow Cytometry

Tumor tissues of mice were obtained and single-cell suspensions were prepared. The cells were blocked with anti-mouse CD16/32 (TruStain fcX™, USA) and identified with anti-mouse CD3, CD4, CD8, and CD45 antibodies (eBioscience). Then single-cell suspension samples were run on a BD FACSVerse™ (BD Biosciences, USA) and analyzed using FlowJo software (TreeStar, USA).

### Bioinformatic Analysis

In this study, GEO (Gene Expression Omnibus) database (https://www.ncbi.nlm.nih.gov/geo/) and TIMER 2.0 web tool (http://timer.cistrome.org/) as well as The GEPIA2 (Gene Expression Profiling Interactive Analysis) web tool (http://gepia.cancer-pku.cn/) were used for bioinformatic analysis. In addition, the dataset comprised mRNA-seq data from 374 TCGA tumors (see TCGA Data Portal at https://tcga-data.nci.nih.gov/tcga/) was used to assess the CCL5/CXCL9-associated lymphocytes infiltration in HCC tumor micro-environment. GSE84005 and GSE25097 were used to evaluate the expression of NLRC3 gene in tumor and its relationship with tumor infiltration of CD8^+^T cells.

### Statistical Analyses

Data were presented as means ± standard deviation (SD) and analyzed with GraphPad Prism 6.0. Significance differences were determined by Student’s t-test or one-way ANOVA using a Tu-key *post hoc*-test. *P*<0.05 was considered statistically significant (*P<0.05, **P<0.01, and ***P<0.001). The χ^2^ test was used for assessing the relationship. Kaplan–Meier analysis and the log-rank test were used for survival analysis. Independent prognostic factors were analyzed by the Cox proportional hazards regression model.

## Results

### NLRC3 Expression Is Reduced in HCC Tissue and Its Correlation With Clinical Features of HCC Patients

**Figure 1:**
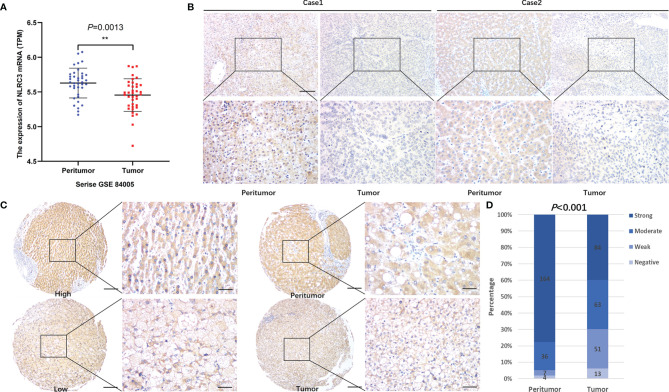
NLRC3 is significantly reduced in HCC tumor tissues. **(A)** Marked reduction of NLRC3 mRNA in the HCC group shown by data from the GSE84005 datasets, data was showed as mean expression intensity +SEM. (**P=0.0013). **(B)** NLRC3 in HCC tissue shown by IHC (magnification 40×, scale bar=500μm. boxed area 400×, scale bar=50μm). **(C)** Representative images of two paired tumor and peritumor tissue samples showing NLRC3 staining (magnification 40×, scale bar=500μm. boxed area 400×, scale bar=50μm). **(D)** 211 paired samples were detected in this study, NLRC3 expression of tumor tissues was significantly lower than paired peritumor tissue, data was showed as mean expression intensity +SEM.

Firstly, NLRC3 expression was performed by bioinformatic analysis with the GSE84005 dataset. It was found NLRC3 mRNA levels were significantly reduced in HCC comparing to peritumor tissues **(** **Figure 1A**). In addition, we assessed the NLRC3 mRNA expression levels in HuH‐7 cells and Hep3B cells by using human monocytic cell line THP‐1 as a positive reference. NLRC3 expression was slightly lower in HuH-7 cells and Hep3B cells compared with THP-1(**Supplementary Figure 1**). So, we followingly verified this relationship by IHC analysis of TMAs with clinical 211 HCC patients, whose clinical and pathologic characteristics were summarized in **Supplementary Table 1**. Paired normal and tumor tissues were acquired from each patient and performed by IHC with NLRC3 antibody. NLRC3 was predominantly located in the cell cytoplasm and shown a clear reduction of NLRC3 expression in HCC tissues (**Figures 1B, C**). Compared with peritumor tissues, NLRC3 expression was reduced in HCC tumor tissues (**Figure 1D**, ***P<0.0001).

According to the expression of NLRC3 in tumor tissues, patients were classified into NLRC3-high group (n = 147) and NLRC3-low group (n = 64). As shown in **Table 1**, raised NLRC3 expression was related to both age (*P*=0.040) and gender (*P*=0.023). However, there is no any significant relationships between NLRC3 and other clinical or pathological characteristics, including tumor differentiation, tumor size or number, TNM stage, Barcelona clinic liver cancer (BCLC) stage, tumor thrombus, tumor capsule, and history of cirrhosis.

**Table 1:** Correlation of clinicopathological parameters and NLRC3 expression.

Clinical characteristic	NLRC3^Low^	NLRC3^High^	*P*-Value
	No.	No.	
**Age (No.)**			0.040*
< 60	45	121	
≥60	19	26	
**Gender**			0.023*
Male	50	132	
Female	14	15	
**Tumor differentiation**			0.360
I-II	48	105	
III-IV	16	42	
**Tumor size (cm)**			0.079
< 5	30	86	
≥5	34	61	
**Tumor number**			0.121
Single	52	130	
Multiple	12	17	
**TNM stage**			0.055
I-II	55	138	
III-IV	9	9	
**BCLC stage**			0.190
O-A	26	71	
B-C	38	76	
**Tumor thrombus**			0.322
No	43	105	
Yes	21	42	
**Tumor capsule**			0.442
No	26	63	
Yes	38	84	
**History of cirrhosis**			0.367
No	11	30	
Yes	53	117	

Bold value and * represent the Statistically significant.

Univariate analysis showed that the NLRC3 level, together with tumor size, and BCLC stage, contributed to HCC outcome. Multivariate analysis was used to assess the independence of NLRC3 in prognosis prediction. After adjustment for the factors identified by the univariate analysis, NLRC3 did not appear to be an independent factor for overall survival (HR=0.773, 95%CI: 0.512-1.165, *P*=0.2181) (**Table 2**) or disease-free survival (HR=0.677, 95%CI: 0.450-1.016, *P*=0.0596) (**Table 3**) in HCC.

**Table 2:** Univariate and multivariate analysis of clinicopathological and NLRC3 for overall survival (OS) in patients with HCC (n = 211).

Variables		Univariate analysis			Multivariate analysis		
		HR	95%Cl	*P*-value	HR	95%Cl	*P*-value
Age	<60 vs.≥60	1.275	0.828-1.963	0.2429	NA		
Gender	Male vs. female	1.476	0.811-2.685	0.2024	NA		
Tumor differentiation	I-II vs. III-IV	1.343	0.903-1.999	0.1457	NA		
Tumor size(cm)	≤5 vs. >5	2.200	1.519-3.187	0.0001*	2.061	1.185-3.585	0.0104*
Tumor capsule	yes vs. no	1.278	0.885-1.846	0.1907	NA		
History of cirrhosis	yes vs. no	1.490	0.972-2.282	0.0671	NA		
BCLC stage	I-II vs. III-IV	2.079	1.419-3.046	0.0002*	1.277	0.716-2.279	0.4071
NLRC3	low vs. high	0.633	0.423-0.951	0.0276*	0.773	0.512-1.165	0.2181

Bold value and * represent the Statistically significant. NA, not adopted; CI, confidence interval; HR, hazard ratio.

**Table 3:** Univariate and multivariate analysis of clinicopathological and NLRC3 for disease-free survival (DFS) in patients with HCC (n = 211).

Variables		Univariate analysis			Multivariate analysis		
		HR	95%Cl	*P*-value	HR	95%Cl	*P*-value
Age	<60 vs.≥60	1.243	0.807-1.915	0.3242	NA		
Gender	male vs. female	1.581	0.869-2.876	0.1337	NA		
Tumor differentiation	I-II vs. III-IV	1.256	0.844-1.864	0.2621	NA		
Tumor size(cm)	≤5 vs. >5	1.813	1.257-2.617	0.0015*	1.843	1.001-3.392	0.0496*
Tumor capsule	yes vs. no	1.239	0.858-1.789	0.2526	NA		
History of cirrhosis	yes vs. no	1.491	0.973-2.286	0.0666	NA		
BCLC stage	I-II vs. III-IV	1.962	1.340-2.871	0.0005*	1.607	0.906-2.851	0.1051
NLRC3	low vs. high	0.666	0.446-0.994	0.0468*	0.677	0.450-1.016	0.0596

Bold value and * represent the Statistically significant. NA, not adopted; CI, confidence interval; HR, hazard ratio.

### High NLRC3 Expression Is Correlated With Favorable Prognosis of OS and DFS in HCC Patients

**Figure 2:**
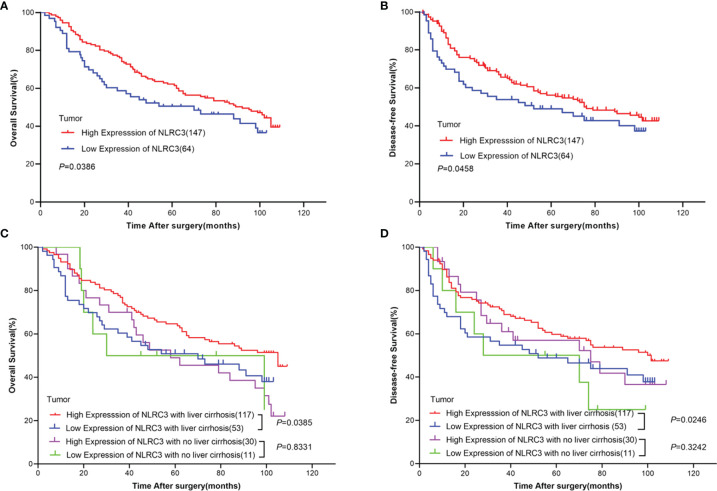
High NLRC3 expression correlated with favorable prognostic in HCC patients. **(A)** The OS in NLRC3 high expression group was higher than NLRC3 low expression group by Kaplan-Meier analysis, (*P*=0.0386). **(B)** The DFS in NLRC3 high expression group was higher than NLRC3 low expression group by Kaplan-Meier analysis, (*P*=0.0458). **(C)** The OS in NLRC3 high expression group was higher than NLRC3 low expression group by Kaplan-Meier analysis in patients with a history of cirrhosis, (*P*=0.0385). **(D)** The DFS in NLRC3 high expression group was higher than NLRC3 low expression group by Kaplan-Meier analysis in patients with a history of cirrhosis, (*P*=0.0246).

The prognostic value of NLRC3 expression was investigated by Kaplan-Meier survival analysis. Interestingly, the results showed NLRC3 high expression was associated with a favorable overall and disease-free survival of HCC patients (**Figures 2A, B**). Because NLRC3 is involved in inflammation response, the liver cirrhosis of HCC patients was taken into account. Same findings were shown in **Figures 2C, D**, high NLRC3 expression also correlated with favorable prognostic of overall survival (OS) and disease-free survival (DFS) in HCC patients with a history of liver cirrhosis. However, in HCC patients group without a history of cirrhosis, there was no difference in OS and DFS between the high and low NLRC3 expression group.

### NLRC3 Expression Is Positively Correlated With CD8^+^ T Cell Infiltration

**Figure 3:**
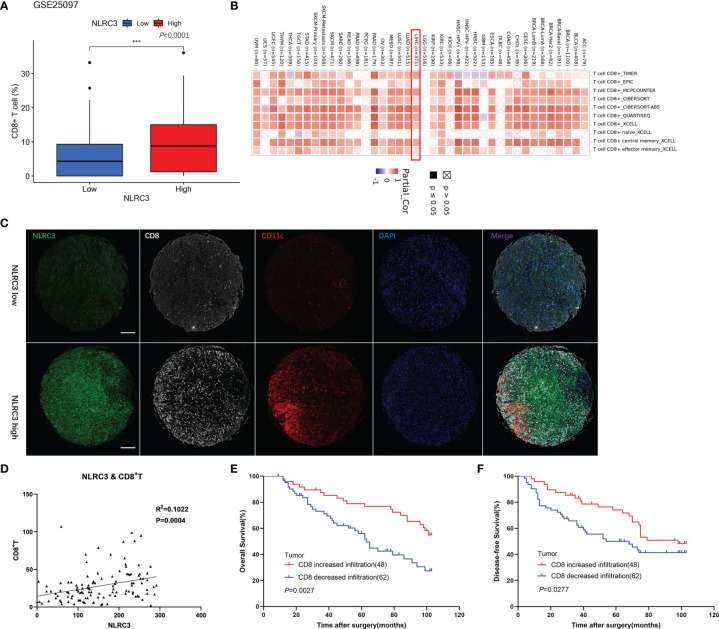
Strong NLRC3 expression in HCC tissue positively correlated with high CD8^+^ T cell infiltration. **(A)** CD8^+^ T cells in high NLRC3 mRNA subgroups was higher than low NLRC3 mRNA subgroups from the GSE25097 datasets, data was showed as mean. (***P<0.0001). **(B)** NLRC3 level and CD8^+^T cell infiltration was positively correlated. **(C)** Representative mIHC images of two tumor tissue samples showing NLRC3, CD8 and CD11c staining in TMAs (magnification 40×, scale bar=500μm). **(D)** NLRC3 expression in HCC tissue positively correlated with CD8^+^ T cell infiltration (*P*=0.0004), data were analyzed by Pearson’s R^2^ correlation test. **(E)** The OS in CD8 increased infiltration group was higher than CD8 decreased infiltration group by Kaplan-Meier analysis (*P*=0.0027). **(F)** The DFS in CD8 increased infiltration group was higher than CD8 decreased infiltration group by Kaplan-Meier analysis (*P*=0.0277).

In order to verify our suppose that NLRC3 maybe links with CD8^+^ T cell infiltration, we firstly analyzed the relationship between CD8^+^ T cell infiltration and NLRC3 expression by bioinformatic analysis with the GSE25097 datasets in GEO database and TIMER 2.0 web tool. The results indicated a higher degree of CD8^+^ T cell infiltration in the subgroups with higher NLRC3 mRNA levels and there was a positive correlation between them, as shown in **Figures 3A, B**. Followingly, bioinformatic data was identified by TMAs of HCC patients. NLRC3 protein levels were investigated by mIHC in HCC tumor tissues on the TMAs, together with CD8 and CD11c infiltration (**Figure 3C**). This showed a significant association between NLRC3 and CD8 expression representing CD8^+^ T cell infiltration (R^2^ = 0.1022, *P* = 0.0004) (**Figure 3D**). Simultaneously, it is accompanied by the expression of CD11c, a surface marker in most antigen-presenting cells (APCs). Kaplan-Meier and log-rank tests showed that high CD8 was a prognosticator for improved overall survival (*P* = 0.0027; **Figure 3E**) and disease-free survival (*P* = 0.0277; **Figure 3F**), indicating that NLRC3 strengthens the prognostic value of CD8^+^ T cells for HCC.

### Knockdown of NLRC3 Inhibits CD8^+^ T Cell Infiltration in the Tumor-Bearing Mouse Models

**Figure 4:**
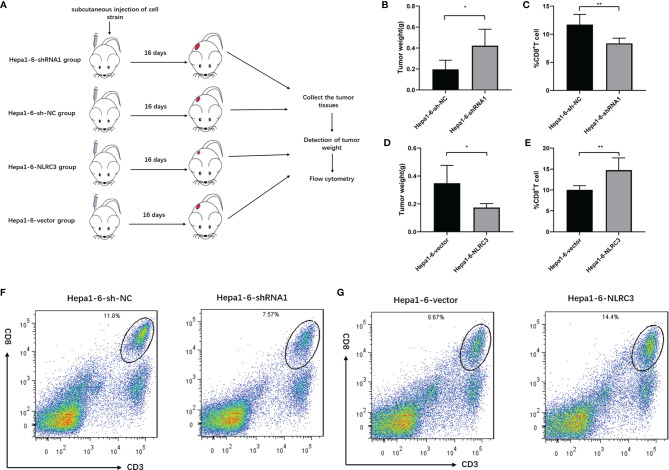
NLRC3 promotes CD8^+^ T cell infiltration in tumor-bearing mouse models. **(A)** Tumor bearing mouse models were divided into 4 groups. **(B)** Compared to Hepa1-6-sh-NC group, the tumor weights were significantly increased in the shRNA1 group (*P=0.0220). **(C)** Compared to Hepa1-6-sh-NC group, the percentages of CD3^+^CD8^+^ T cells were significantly decreased in the shRNA1 group (n = 5) (**P=0.0053). **(D)** Compared to Hepa1-6-vector group, the tumor weights were significantly decreased in the Hepa1-6-NLRC3 group (*P=0.0178). **(E)** Compared to Hepa1-6-vector group, the percentages of CD3^+^CD8^+^ T cells were significantly increased in the Hepa1-6-NLRC3 group (n = 5) (**P=.0094). **(F)** Representative Flow cytometry analysis of CD8^+^ T cells on day 16 showing percentages of CD3^+^CD8^+^ T cells in the Hepa1-6-sh-NC and Hepa1-6-shRNA1 groups. **(G)** Representative Flow cytometry analysis of CD8^+^ T cells on day 16 showing percentages of CD3+CD8+ T cells in Hepa1-6-vector and Hepa1-6-NLRC3 group.

To further determine the effect of NLRC3 on CD8^+^ T cell infiltration in the tumor-bearing mouse models, we established the Hepa1-6-sh-NC-bearing and Hepa1-6-shRNA1-bearing models and analyzed them by FACs on day 16 (as shown in **Figure 4A**). We found tumor weight increased in Hepa1-6-shRNA1-bearing mice group (**Figure 4B**) and there were significantly fewer CD3^+^CD8^+^ T cells present among infiltrating immune cells (CD45^+^ live cells) in Hepa1-6-shRNA1 tumors compared to Hepa1-6-sh-NC tumors (**Figure 4C**). Consistently, tumor weight was decreased (**Figure 4D**) and CD3^+^CD8^+^ T cells were increased in Hepa1-6-NLRC3-bearing group (**Figure 4E**). Representative Flow cytometry analysis of CD8^+^ T cell infiltration was shown in **Figures 4F, G**. The NLRC3 gene expression efficiency of the NLRC3-overexpression/know-down cell lines used in this experiment has been verified (**Supplementary Figure 2**). This suggests that NLRC3 promotes the CD8^+^ T cell infiltration *in vivo* and inhibits the tumor growth.

### NLRC3 Positively Correlates With CCL5 and CXCL9 Expressions

**Figure 5:**
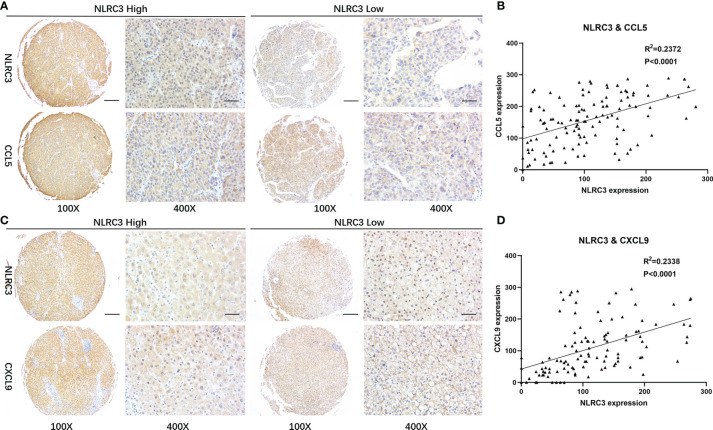
Correlations between NLRC3 and T-cell recruiting chemokines. **(A)** Representative images of NLRC3 and CXCL9 expression in HCC detected by IHC on the TMAs (magnification 40×, scale bar=500μm. boxed area 400×, scale bar=50μm). **(B)** NLRC3 expression in HCC tissue positively correlated with CCL5 expression (*P*<0.0001), data were analyzed by Pearson’s R^2^ correlation test. **(C)** Expressions of NLRC3 and CXCL9 in HCC (magnification 40×, scale bar=500μm. boxed area 400×, scale bar=50μm). **(D)** NLRC3 expression in HCC tissue positively correlated with CXCL9 expression (*P*<0.0001), data were analyzed by Pearson’s R^2^ correlation test.

A previous study reported that CD8^+^T cell infiltration in tumors is associated with CCL5 and CXCL9 co-expression (14). Our results of the bioinformatics analysis show that CCL5 and CXCL9 were positively correlated with the content of T cell CD8+、T cell、B cell、NK cell and macrophage lymphocytes in the liver tumor micro-environment (**Supplementary Figure 3**). To explore whether these chemokines also play certain role in NLRC3 higher expression and CD8^+^ T cell infiltration, the results of the bioinformatics reveal that NLRC3 was positively correlated with chemokine CCL5 and CXCL9 in tumor at mRNA level (**Supplementary Figure 4**). In order to validate these finding, immunohistochemistry assay on the TMAs of HCC patients were performed. The data revealed that CCL5 is expressed in HCC tumor tissues and its expression is significantly correlated with NLRC3 (*P*<0.0001, R^2^ = 0.2372) (**Figures 5A, B**). At the same time, it was also confirmed that NLRC3 was positively correlated with CXCL9 (*P*<0.0001, R^2^ = 0.2338) expressions in HCC tumor (**Figures 5C, D**). These results further suggest that NLRC3 may promote the infiltration of CD8^+^T cells into the HCC tumor microenvironment by mediating chemokines CCL5 and CXCL9.

## Discussion

HCC is extremely common and causes the second highest number of cancer-associated deaths worldwide and some studies have revealed that inflammation is one of the main driving forces for its progression (15). As we know, tumor microenvironmental regulated the tumor progression and metastasis (16). Persistent inflammatory responses can alter several components of the tumor microenvironment, including the increase of inflammatory cytokines, such as TNF-α、IL-8 (17, 18). So, for patients with HCC, the occurrence of persistent inflammatory reaction in liver tissues will lead to high recurrence rate and extremely significant invasiveness. In the tumor microenvironment, as an important part of the inflammatory response, NLR family proteins and chemokines as well as infiltrated immune cells play important role in tumor development (19). As an important member of the NLR family, NLRC3 negatively regulates inflammation and suppresses it occurrence in various tissues (20). We screened and analyzed data from large bioinformatics databases, finding that NLRC3 levels differ between HCC and normal tissues. However, the significance of NLRC3 in HCC clinical management and its involvement in tumor immunology remain unclear. Interestingly, the involvement of NLRC3 in the malignant progression of other tumors has been widely reported. In colorectal cancer, it has been shown thatNLRC3 is significantly down-regulated and these patients have a poorer survival prognosis. This reveals that NLRC3 has a potential tumor suppression effect in the malignant progression of colorectal cancer (21). This is similar to mouse model findings where it was found that NLRC3 knockouts are more likely to develop colorectal inflammation and colorectal cancer (22). Therefore, NLRC3 may be an important factor affecting the malignant progression of liver cancer.

We investigated the relationship between NLRC3 levels in cancer tissues and patient prognosis based on histochemical staining of TMA tumor samples, clinicopathological data, and survival data of 211 HCC patients. This showed that HCC patients with high NLRC3 levels in cancer tissues had a favorable survival prognosis. Notably, it was observed that raised NLRC3 levels in HCC patients with a history of liver cirrhosis were linked to both overall and disease-free survival. Since cirrhosis is accompanied by damage and chronic inflammation in liver tissue (23). NLRC3 can effectively control the inflammatory response by negatively regulating the NF-κB signaling pathway and coordinating the interaction between caspase 1, IL-1β, and IL-18 expression, suggesting that NLRC3 may further increase the survival of HCC patients with a history of liver cirrhosis by reducing inflammation (24, 25). In subsequent single-factor and multi-factor analysis, it was found that the NLRC3 level represents an independent prognostic indicator for liver cancer. Thus, not only does NLRC3 suppress the malignant progression of liver cancer but its level in cancer tissue can be used as a measure of malignancy in HCC.

Although the results of this part of the study concluded that NLRC3 suppresses the progression of liver tumors, the mechanism responsible for this effect was still not clear. This required further testing using HCC clinical specimens combined with animal experiments for verification. Therefore, the next part of the study investigated the relationship between NLRC3 and CD8^+^ T cell infiltration in liver tumor microenvironments from the perspective of tumor immunology. Studies have shown that NLRC3 can inhibit systemic inflammation, reduce the ratio of neutrophils to lymphocytes in tissues, and increase the number of lymphocytes in the tissue microenvironment (26). In addition, as a major component of intracellular anti-inflammatory proteins, NLRC3 can negatively regulate inflammatory signaling pathways, reduce the release of inhibitory immune regulatory mediators, and increase the secretion of chemokines related immune cell infiltration into the TME, thereby causing the increase of anti-tumor lymphocytes in the body tissues (27). In colorectal cancer, NLRC3 inhibits systemic inflammation, leading to a decrease in NLR value and an increase in anti-tumor lymphocytes-cytotoxic T lymphocytes (28). Thus, the relationship between NLRC3 and lymphocytes might explain the promotion of CD8^+^ T cell infiltration by NLRC3. And the subtype of infiltrating CD8^+^ T cell maybe cytotoxic T lymphocytes.

As a cytokine secreted by T lymphocytes, macrophages, and some other cells, CCL5 can regulate the expression and secretion of normal T cells and bind with CCR5 (C-C chemokine receptor type 5) to recruit anti-tumor T cells and dendritic cells to TME (27). CCL5 induced tumor immune tolerance by recruiting and regulating the activity of inflammatory cells such as T cells (29). It was found that after the expression of CCL5 was blocked by gene knockout, the recruitment of Tregs into the tumor microenvironment was slowed, thus effectively reducing the proliferation of tumor cells (30). Another myeloid cell-secreted chemokine CXCL9 correlates the engraftment of tumor-infiltrating lymphocytes and amplifies the anti-tumor response of cytotoxic T lymphocytes (14, 31). It was highly expressed in malignant tumors and promotes the infiltration of CD4^+^ and CD8^+^ lymphocytes into tumor cell regions to kill tumor cells (32). CCL5 and CXCL9 were positively correlated with CD8^+^T cell infiltration in solid tumors. Previous studies have found that when cancer cells reduce CCL5 production, CXCL9 expression also decreased. Cancer cells can inhibit CCL5 expression because it attracted CD8^+^T cells (33). The researches showed when CCL5 attracted T cells to the tumor and activated cancer antigens. Then it released its own signaling protein, IFN-γ (interferon γ). Macrophages and dendritic cells that cluster on the tumor secrete CXCL9, which significantly promoted tumor invasion through circulating T cells (34). Therefore, CCL5 is a key chemokine that determines whether a tumor will be infiltrated by T cells. However, CCL5 expression alone is not sufficient, as CXCL9 is the primary amplifier for T cell recruitment. Our study firstly revealed the positive correlations between NLRC3 and CCL5 and CXCL9 expressions in HCC, which explained the mechanisms of NLRC3-related CD8^+^ T cell infiltration in tumor.

In a conclusion that NLRC3 may suppress HCC progression through promoting CD8^+^ T cells infiltration by CCL5 and CXCL9. This study also implies that boosting NLRC3 is a promising strategy to inhibit the development of tumor and thus increase the survival of HCC patients in clinic hospital.

## Data Availability Statement

The original contributions presented in the study are included in the article/**Supplementary Material**. Further inquiries can be directed to the corresponding authors.

## Ethics Statement

The studies involving human participants were reviewed and approved by The ethics committee of Zhongshan Hospital, Fudan University. The patients/participants provided their written informed consent to participate in this study. The animal study was reviewed and approved by The ethics committee of Shanghai Medical College, Fudan University.

## Author Contributions

CW, JS, and JX contributed equally to this work for performing the experiments as well as the preparation for the manuscript. JY, QF, and YD performed the IHC of tumor tissue microarrays (TMAs). BL helped for the analysis of clinical data. QG, JQ, and CL design the research together and prepare for the manuscript. All authors contributed to the article and approved the submitted version.

## Funding

This study was supported by the National Natural Science Foundation of China (grant no. 31971111).

## Conflict of Interest

The authors declare that the research was conducted in the absence of any commercial or financial relationships that could be construed as a potential conflict of interest.

## Publisher’s Note

All claims expressed in this article are solely those of the authors and do not necessarily represent those of their affiliated organizations, or those of the publisher, the editors and the reviewers. Any product that may be evaluated in this article, or claim that may be made by its manufacturer, is not guaranteed or endorsed by the publisher.

## Abbreviations

BCLC, Barcelona clinic liver cancer; CCL5, C-C motif chemokine ligand 5; CXCL9, C-X-C motif chemokine ligand 9; DFS, disease-free survival; GEO, gene expression omnibus; GEPIA, gene expression profiling interactive analysis; HCC, hepatocellular carcinoma; IHC, immunohistochemistry; mIHC, multiple immunohistochemistry; NLRC3, NLR family caspase recruitment domain containing 3; OS, overall survival; TIMER, tumor immune estimation resource; TMAs, tissue microarrays; TME, tumor microenvironment.
