# Supplementary material for: NLRC3 High Expression Represents a Novel Predictor for Positive Overall Survival Correlated With CCL5 and CXCL9 in HCC Patients
Source: Front Oncol. 2022 Jan 25;12:815326. doi: 10.3389/fonc.2022.815326 (PMC8821914; doi:10.3389/fonc.2022.815326)
Supplement: Supplementary file 1 [file DataSheet_1.docx]

Supplementary Material

# Supplementary Table

**Supplementary Table 1. Clinical characteristics of the 211 HCC patients in this study**

| **Variables** | **Number** | **Percentage (%)** |
| --- | --- | --- |
| **Age(years)** |  |  |
| < 60 | 166 | 78.7 |
| ≥60 | 45 | 21.3 |
| **Gender** |  |  |
| Male | 182 | 86.3 |
| Female | 29 | 13.7 |
| **Tumor differentiation** |  |  |
| I-II | 153 | 72.5 |
| III-IV | 58 | 27.5 |
| **Tumor size(cm)** |  |  |
| < 5 | 116 | 55 |
| ≥5 | 95 | 45 |
| **Tumor number** |  |  |
| single | 182 | 86.3 |
| multiple | 29 | 13.7 |
| **TNM stage** |  |  |
| I-II | 193 | 91.5 |
| III-IV | 18 | 8.5 |
| **BCLC stage** |  |  |
| O-A | 97 | 46.0 |
| B-C | 114 | 54.0 |
| **Tumor thrombus** |  |  |
| No | 148 | 70.1 |
| Yes | 63 | 29.9 |
| **Tumor capsule** |  |  |
| No | 122 | 57.8 |
| Yes | 89 | 42.2 |
| **History of cirrhosis** |  |  |
| No | 41 | 19.4 |
| Yes | 170 | 80.6 |
|  |  |  |

2. **Supplementary Figure 1**


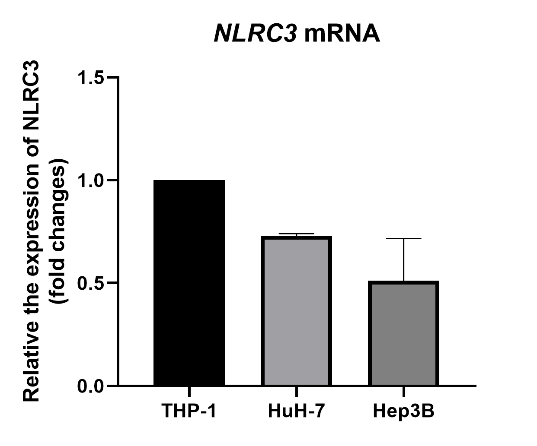


**Supplementary Figure 1**. RT‐PCR analysis of NLRC3 expression in THP-1、HuH‐7 and Hep3B cells.

3. **Supplementary Figure 2**


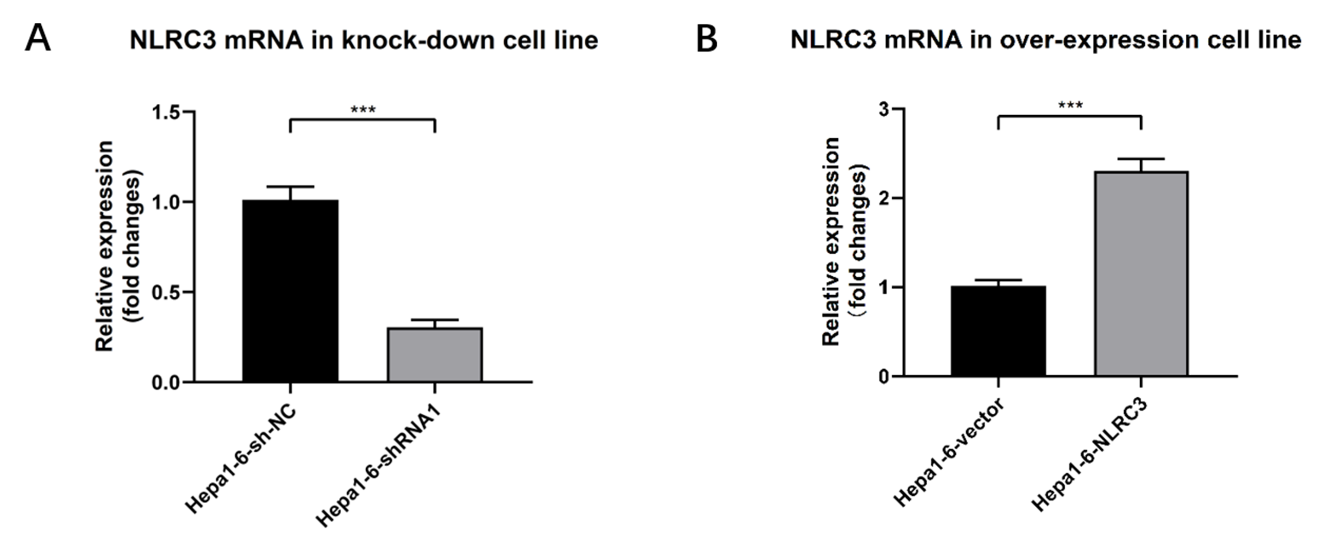


**Supplementary Figure 2**. Validation results of NLRC3-overexpression/know-down cell lines. **(A)** Relative levels of NLRC3 mRNA in Hepa1-6-shRNA1 and Hepa1-6-sh-NC detected by RT-PCR. **(B)** Relative levels of NLRC3 mRNA in Hepa1-6-NLRC3 and Hepa1-6-vector detected by RT-PCR.

4. **Supplementary Figure 3**


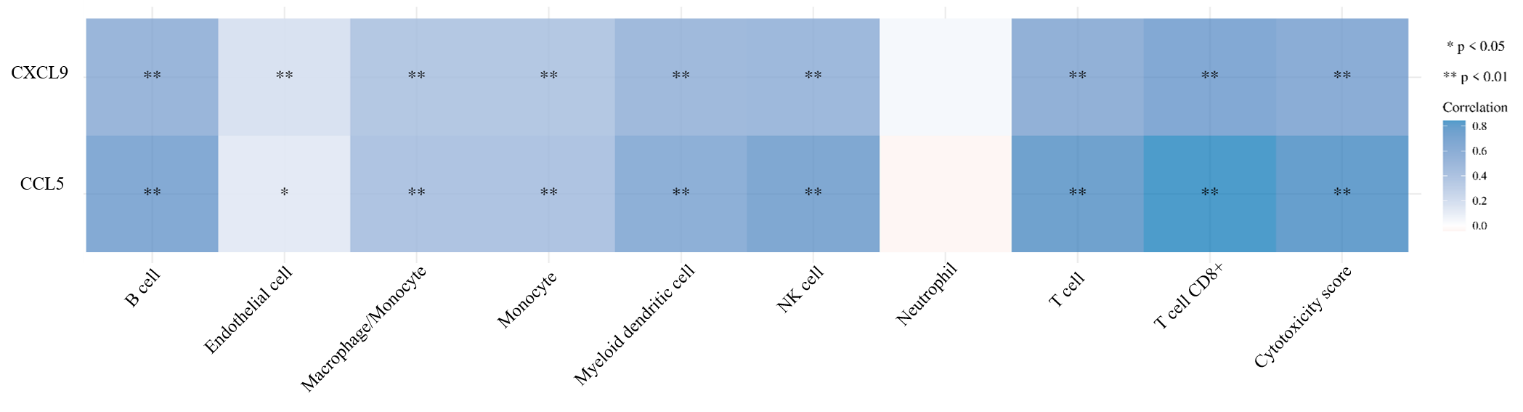


**Supplementary Figure 3**. CCL5/CXCL9-associated lymphocytes infiltration in HCC tumor micro-environment

4. **Supplementary Figure 4**


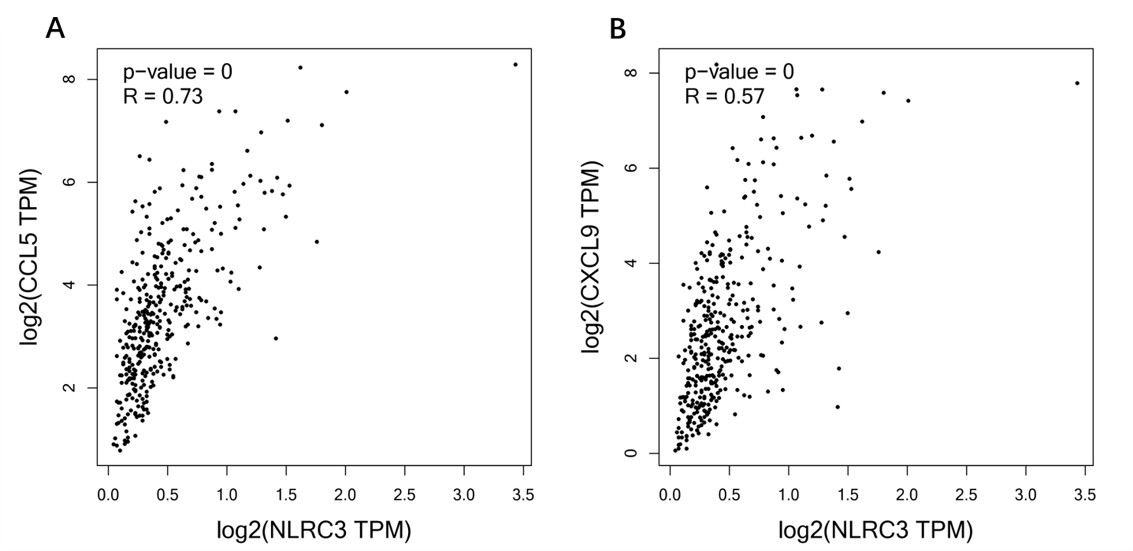


**Supplementary Figure 4**. Correlation between NLRC3 and chemokines (CCL5 and CXCL9) in HCC predicted by GEPIA2. **(A)** NLRC3 and CCL5 mRNA expressions in tumor analyzed by Pearson correlation coefficient. **(B)** NLRC3 and CXCL9 mRNA expressions in tumor analyzed by Pearson correlation coefficient.
